# Supplementary material for: Modeling glioblastoma heterogeneity as a dynamic network of cell states
Source: Mol Syst Biol. 2021 Sep 16;17(9):e10105. doi: 10.15252/msb.202010105 (PMC8444284; doi:10.15252/msb.202010105)
Supplement: Supplementary file 6 — Source Data for Figure 5 [file MSB-17-e10105-s004.zip › Figure5A_sourcedata/GSEA_3017/hallmarks_stateD.GseaPreranked.1621934542601/HALLMARK_HYPOXIA.html]

Details for gene set HALLMARK\_HYPOXIA[GSEA]

|  || Dataset | state23017 |
| Phenotype | NoPhenotypeAvailable |
| Upregulated in class | na\_neg |
| GeneSet | HALLMARK\_HYPOXIA |
| Enrichment Score (ES) | -0.29625034 |
| Normalized Enrichment Score (NES) | -1.3068134 |
| Nominal p-value | 0.16195652 |
| FDR q-value | 0.27128902 |
| FWER p-Value | 0.996 |
Table: GSEA Results Summary

  

Fig 1: Enrichment plot: HALLMARK\_HYPOXIA      
 Profile of the Running ES Score & Positions of GeneSet Members on the Rank Ordered List

  

| PROBE | GENE SYMBOL | GENE\_TITLE | RANK IN GENE LIST | RANK METRIC SCORE | RUNNING ES | CORE ENRICHMENT || 1 | MIF |  |  | 48 | 0.351 | -0.0126 | No |
| 2 | BTG1 |  |  | 50 | 0.350 | 0.0112 | No |
| 3 | STC1 |  |  | 67 | 0.322 | 0.0215 | No |
| 4 | LDHA |  |  | 92 | 0.297 | 0.0236 | No |
| 5 | MAP3K1 |  |  | 103 | 0.289 | 0.0362 | No |
| 6 | PPP1R15A |  |  | 149 | 0.259 | 0.0194 | No |
| 7 | GPC1 |  |  | 213 | -0.255 | -0.0117 | No |
| 8 | NAGK |  |  | 400 | -0.273 | -0.1371 | No |
| 9 | BNIP3L |  |  | 431 | -0.277 | -0.1409 | No |
| 10 | GCNT2 |  |  | 492 | -0.284 | -0.1676 | No |
| 11 | NEDD4L |  |  | 595 | -0.299 | -0.2259 | No |
| 12 | CCNG2 |  |  | 623 | -0.305 | -0.2255 | No |
| 13 | PGM1 |  |  | 715 | -0.322 | -0.2736 | Yes |
| 14 | SDC3 |  |  | 744 | -0.329 | -0.2722 | Yes |
| 15 | PFKL |  |  | 771 | -0.337 | -0.2687 | Yes |
| 16 | ANXA2 |  |  | 777 | -0.338 | -0.2489 | Yes |
| 17 | IDS |  |  | 782 | -0.339 | -0.2281 | Yes |
| 18 | MYH9 |  |  | 820 | -0.350 | -0.2323 | Yes |
| 19 | TIPARP |  |  | 841 | -0.355 | -0.2229 | Yes |
| 20 | DTNA |  |  | 857 | -0.359 | -0.2093 | Yes |
| 21 | CDKN1B |  |  | 862 | -0.359 | -0.1871 | Yes |
| 22 | GPC4 |  |  | 891 | -0.369 | -0.1829 | Yes |
| 23 | SAP30 |  |  | 905 | -0.376 | -0.1666 | Yes |
| 24 | RRAGD |  |  | 928 | -0.386 | -0.1565 | Yes |
| 25 | WSB1 |  |  | 961 | -0.398 | -0.1534 | Yes |
| 26 | PDGFB |  |  | 969 | -0.401 | -0.1306 | Yes |
| 27 | AKAP12 |  |  | 994 | -0.412 | -0.1203 | Yes |
| 28 | VLDLR |  |  | 1004 | -0.416 | -0.0981 | Yes |
| 29 | PAM |  |  | 1055 | -0.448 | -0.1054 | Yes |
| 30 | CHST2 |  |  | 1081 | -0.460 | -0.0925 | Yes |
| 31 | SDC2 |  |  | 1200 | -0.580 | -0.1435 | Yes |
| 32 | SERPINE1 |  |  | 1218 | -0.617 | -0.1133 | Yes |
| 33 | F3 |  |  | 1241 | -0.673 | -0.0831 | Yes |
| 34 | CSRP2 |  |  | 1298 | -0.986 | -0.0572 | Yes |
| 35 | CDKN1A |  |  | 1306 | -1.046 | 0.0109 | Yes |
Table: GSEA details [plain text format]

  

Fig 2: HALLMARK\_HYPOXIA: Random ES distribution      
 Gene set null distribution of ES for **HALLMARK\_HYPOXIA**
